# Vps28 Is Involved in the Intracellular Trafficking of Awd, the *Drosophila* Homolog of NME1/2

**DOI:** 10.3389/fphys.2019.00983

**Published:** 2019-08-02

**Authors:** Elisa Mezzofanti, Marilena Ignesti, Tien Hsu, Giuseppe Gargiulo, Valeria Cavaliere

**Affiliations:** ^1^Dipartimento di Farmacia e Biotecnologie, Alma Mater Studiorum – Università di Bologna, Bologna, Italy; ^2^Department of Biomedical Sciences and Engineering, National Central University, Zhongli, Taiwan; ^3^Center for Chronic Disease Management and Research, National Central University, Zhongli, Taiwan

**Keywords:** Awd/NME, metastasis suppressor genes, intracellular trafficking, ESCRT machinery, Vps28, ALiX, *Drosophila*, fat body

## Abstract

The *Awd* (*abnormal wing discs*) gene is the *Drosophila* homolog of human *NME1* and *NME2* metastasis suppressor genes. These genes play a key role in tumor progression. Extensive studies revealed that intracellular NME1/2 protein levels could be related to either favorable or poor prognosis depending on tissue context. More recently, extracellular activities of NME1/2 proteins have also been reported, including a tumor- promoting function. We used *Drosophila* as a genetic model to investigate the mechanism controlling intra- and extracellular levels of NME1/2. We examined the role of several components of the ESCRT (endosomal sorting complex required for transport) complex in controlling Awd trafficking. We show that the Vps28 component of the ESCRT−I complex is required for maintenance of normal intracellular level of Awd in larval adipocytes. We already showed that blocking of Shibire (Shi)/Dynamin function strongly- lowers Awd intracellular level. To further investigate this down regulative effect, we analyzed the distribution of endosomal markers in wild type and Shi-defective adipocytes. Our results suggest that Awd does not enter CD63-positive endosomes. Interestingly, we found that in fat body cells, Awd partly- colocalizes with the ESCRT accessory component ALiX, the ALG-2 (apoptosis-linked gene 2)-interacting protein X. Moreover, we show that the intracellular levels of both proteins are downregulated by blocking the function of the Dynamin encoded by the *shibire* gene.

## Introduction

*NME1* and *NME2* genes are closely related members of the *NME* gene family which consists of 10 members (Stafford et al., 2008). *NME1* was the 1st metastasis suppressor gene identified (Steeg et al., 1988) and together with *NME2* is mostly implicated in tumor progression. Several studies showed that NME1/2 intracellular content is correlated with either lowered or enhanced metastatic abilities depending on tissue context of the developing tumor (Steeg et al., 1993; Okabe-Kado et al., 1998; van Noesel and Versteeg, 2004; Tschiedel et al., 2008). Recently, a correlation between extracellular NME protein level and tumor progression has also been reported in a number of tumor types (Romani et al., 2018).

The *Drosophila Awd* gene is the fly ortholog of *NME1/2* genes (Rosengard et al., 1989). Our studies showed that Awd is an endocytic mediator that interacts with Rab5 and the *Drosophila* homolog of Dynamin1 encoded by the *shibire* (*shi*) gene (Dammai et al., 2003; Nallamothu et al., 2008; Woolworth et al., 2009; Ignesti et al., 2014). We have shown that the larval fat body, among several larval tissues, is able to secrete the Awd protein in the hemolymph (Romani et al., 2016). In addition, we found that Shi controls the balance of Awd intracellular-extracellular levels. While blocking of Shi function in adipocytes leads to downregulated Awd intracellular level, loss of Rab5 function does not. Thus, our results suggest the specific involvement of Shi in the internalization process of Awd molecules circulating in the hemolymph, and the endocytic vesicles containing Awd are sorted into the adipocytes via a Rab5-independent pathway (Romani et al., 2016).

Proteomic studies showed the presence of Awd within extracellular vesicles released in the culture medium by two different *Drosophila* cell lines (Koppen et al., 2011). Extracellular vesicles mediate cellular communication and are involved in numerous biological functions (Christ et al., 2017). These small vesicles can derive from cells of different nature and form throughout different developmental processes (van Niel et al., 2018).

The ESCRT machinery is a membrane remodeling system that acts in a variety of biological processes (Christ et al., 2017). It consists of three multi-subunit complexes ESCRT–I, –II and –III that are recruited by specific targeting molecules. Multivesicular body (MVB) formation requires the ESCRT–0 function to recruit the ESCRT machinery. The four ESCRT complexes, together with additional components, act sequentially to deliver cargoes into the intraluminal vesicles (ILVs). These ILVs could then be released as exosomes in the extracellular space upon fusion of the MVBs with plasma membrane.

The well characterized role of ESCRT in biogenesis and secretion of extracellular vesicles (Christ et al., 2017) sparked our interest in the possibility that the ESCRT machinery might be involved in Awd trafficking. Furthermore, we have recently shown that, in larval wing discs, downregulation of Awd levels coupled with blocking apoptosis causes aneuploidy (Romani et al., 2017) probably due to mitotic defects that have been described in *awd* mutant larval brains (Biggs et al., 1990). The ESCRT complex plays a key role in membrane scission at the end of cytokinesis (Christ et al., 2017) and the ALiX accessory component is required for both completion of abscission (Eikenes et al., 2015) and orientation of the mitotic spindle (Malerød et al., 2018). ALiX is also involved in the biogenesis of exosomes and in the sorting of some cargoes inside these vesicles (Baietti et al., 2012; Hurley and Odorizzi, 2012; Juan and Furthauer, 2018).

In this study, we investigate the involvement of ESCRT machinery in Awd trafficking. We use MARCM system (Lee and Luo, 1999) to generate clones of adipocytes that are null mutants in genes belonging to ESCRT–0, ESCRT–I, ESCRT–II and ESCRT–III complexes. Our analyses of the amount and distribution of intracellular Awd in mutant adipocytes highlight a role of Vps28 component of ESCRT–I complex in controlling the Awd presence inside the cell. In addition, we found that Awd colocalizes with the ALiX accessory component and that Shi function is required for normal intracellular amount of both proteins.

## Results

### Role of ESCRT Machinery in Regulating Awd Intracellular Amount

**FIGURE 1:**
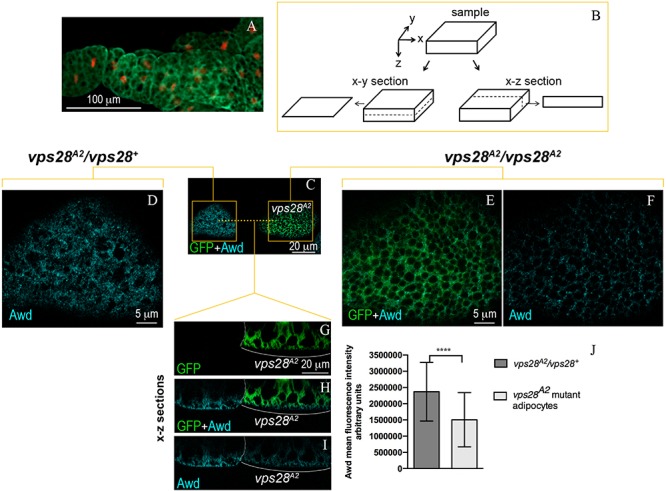
Intracellular distribution of Awd in larval fat body. **(A)** Epifluorescence image of Awd protein expression (green) in fat body dissected from stage L3 larvae carrying the His2Av-mRFP transgene whose expression marks DNA (red). **(B)** Schematic representation of confocal optical sections of a sample in x-y and x-z planes. **(C–I)** Confocal microscopy analysis of *vps28*^*A2*^ MARCM clones marked by GFP expression (**C,E**,**G**,**H**, green) stained for Awd (**C,D**,**F**,**H**,**I**, cyan). **(C)** Surface section (x–y) of mosaic fat body showing a control heterozygous *vps28*^*A2*^/*vps28*^+^ and a mutant *vps28*^*A2*^/*vps28*^*A2*^ adipocyte marked by GFP expression. **(D)** Magnified view of the control *vps28*^*A2*^/*vps28*^+^ adipocyte (left orange box in **C**). **(E,F)** Magnified view of the mutant *vps28*^*A2*^/*vps28*^*A2*^ adipocyte (right orange box in **C**). **(G**–**I)** x–z sections of the adipocytes through the planes indicated by the orange dashed line in **C**. The white dotted line outlines the boundary between the control and mutant adipocyte. The white bracket indicates the mutant adipocyte. **(J)** A severe and significant decrease of Awd signal (*p* = 0.000085) is detectable in *vps28*^*A2*^/*vps28*^*A2*^ adipocyte. Graphs represent mean ± SD amount of Awd in arbitrary units; *n* = 7; ^∗∗∗∗^*p* < 0.0001 (two-tailed, paired *t-*test).

The Awd protein is expressed over all the larval fat body that, in 3rd instar larval stage, consists of layers of tightly connected polygonal adipocytes (Figure 1A). Clones of larval adipocytes homozygous for null mutations of ESCRT genes were generated using the MARCM system (Lee and Luo, 1999). Then, the intracellular localization of Awd was analyzed using immunofluorescence confocal microscopy of fat bodies (see Figure 1B for a scheme of x-y and x-z optical sections).

We started our analysis by looking at the ESCRT–0 complex. In *hrs*^*D28*^, *stam*^2L2896^ double mutant adipocytes, that lack the functions of both genes (Lloyd et al., 2002; Chanut-Delalande et al., 2007), Awd level is unaltered in comparison with its level in wild type adipocytes (Supplementary Figures S1A–C’,L).

We then investigated the role of the ESCRT–I complex. Among the four ESCRT–I subunits, we analyzed the effect of Tsg101 and Vps28 components. Absence of Tsg101 function in clones of adipocytes homozygous for the *tsg101*^2^ loss-of-function allele (Vaccari et al., 2009) does not alter intracellular Awd distribution (Supplementary Figures S1D–K’,M). In wild type cells, Tsg101 mediates recruitment of ESCRT–I to ESCRT–0 complex through direct interaction with Hrs (Christ et al., 2017). The absence of Tsg101 function causes the accumulation of large vesicles, positive for Hrs, which stall along the endocytic pathway. Despite accumulation of the Hrs protein, cells lacking Tsg101 function show normal intracellular profile of Awd (Supplementary Figures S1D–K’,M), further confirming that Hrs is not involved in Awd trafficking.

We then analyzed the effect of Vps28 loss of function in MARCM clones homozygous for the *vps28*^*A2*^ mutation (Vaccari et al., 2009). Surprisingly, immunofluorescence detection of Awd protein in *vps28*^*A2*^ mutant clones (Figures 1C,E,F,H,I) shows a clear downregulation of protein level, when compared to the wild type flanking cells (Figures 1D,G–J). The analysis of *vps28*^*A2*^ mutant adipocytes also shows that the small amount of Awd protein is detectable in its normal subcortical localization indicating that the spatial distribution of Awd is unaltered (Figures 1H,I).

We extended our analysis of Awd trafficking to theVps22 ESCRT–II component (Christ et al., 2017). *vps22*^*ZZ13*^ homozygous adipocytes lacking the function of Vps22 (Vaccari et al., 2009) show an intracellular profile of Awd comparable to that present in the control flanking cells (Supplementary Figures S2A–G,K). Thus, at least this subunit of the ESCRT–II complex is not involved in modulation of the intracellular levels of Awd.

Finally, we analyzed the Vps2 component of the ESCRT–III complex (Christ et al., 2017) by taking advantage of the loss-of-function allele *vps2*^*PP6*^ (Vaccari et al., 2009). In comparison with wild type adipocytes, *vps2*^*PP6*^ homozygous mutant cells show the same intracellular level and the same subcortical distribution of Awd (Supplementary Figures S2H–J’,L). This suggests that the Vps2 component of the ESCRT–III complex is not involved in the regulation of Awd trafficking.

### Analysis of the Endosomal Trafficking in Adipocytes Lacking Shi Function

We have already shown that absence of Shi function causes downregulation of Awd intracellular level in adipocytes coupled with the enhancement of the level of this protein in larval circulating hemolymph (Romani et al., 2016). Moreover, proteomic studies showed that the Awd protein is secreted within extracellular vesicles (Koppen et al., 2011). To further investigate Awd traffic inside and outside cells, we analyzed the effect of defective Shi function on endosomal compartments involved in the secretory pathway.

The exosomes are a particular type of extracellular vesicles that are released by fusion of MVBs, containing the ILVs, with the plasma membrane. The CD63 protein, belonging to the tetraspanine family, is commonly used as an exosome marker. In human cells, this protein is enriched in the ILVs and in the exosomes derived from them (Escola et al., 1998; Wubbolts et al., 2003). Beside biogenesis of exosomes, CD63 is involved in endosomal sorting and in cargo targeting to exosomes (van Niel et al., 2018).

In *Drosophila* cells, it has been shown that the CD63: GFP transgenic protein, consisting of the CD63 heterologous protein fused with GFP, can be used as a marker for MVBs (Panakova et al., 2005). We applied the Flp–Out technique (Pignoni and Zipursky, 1997) to obtain clones of adipocytes expressing CD63:GFP (Supplementary Figures S3A,A’). We found that the Awd protein (Supplementary Figures S3B,B’) does not colocalize with the CD63:GFP chimeric protein (Supplementary Figures S3C,C’). We then asked if blocking Shi function could cause the Awd downregulation by pushing its release through CD63:GFP positive exosomes. We took advantage of the Shi^*K*44*A*^ (Shi^DN^) dominant negative form of Shi whose expression leads to the blocking of Shi activity (Moline et al., 1999). We induced clones of adipocytes coexpressing Shi^DN^ and CD63:GFP proteins through flp–out technique (Supplementary Figures S3D,D’). Shi-defective adipocytes show an increase in the number and size of vesicles positive for CD63:GFP (Supplementary Figures S3D,D’). In *Drosophila* wing discs CD63:GFP marks late endosomes (Panakova et al., 2005). Since Shi^DN^ adipocytes accumulate Rab7-positive late endosomes (Fang et al., 2016), it is possible that these late endosomes accumulate CD63:GFP. The analysis of Awd distribution (Supplementary Figures S3E,E’,F,F’) shows that even within Shi-defective adipocytes there is no colocalization of Awd and CD63:GFP.

### Awd Partially Colocalizes With ALiX

**FIGURE 2:**
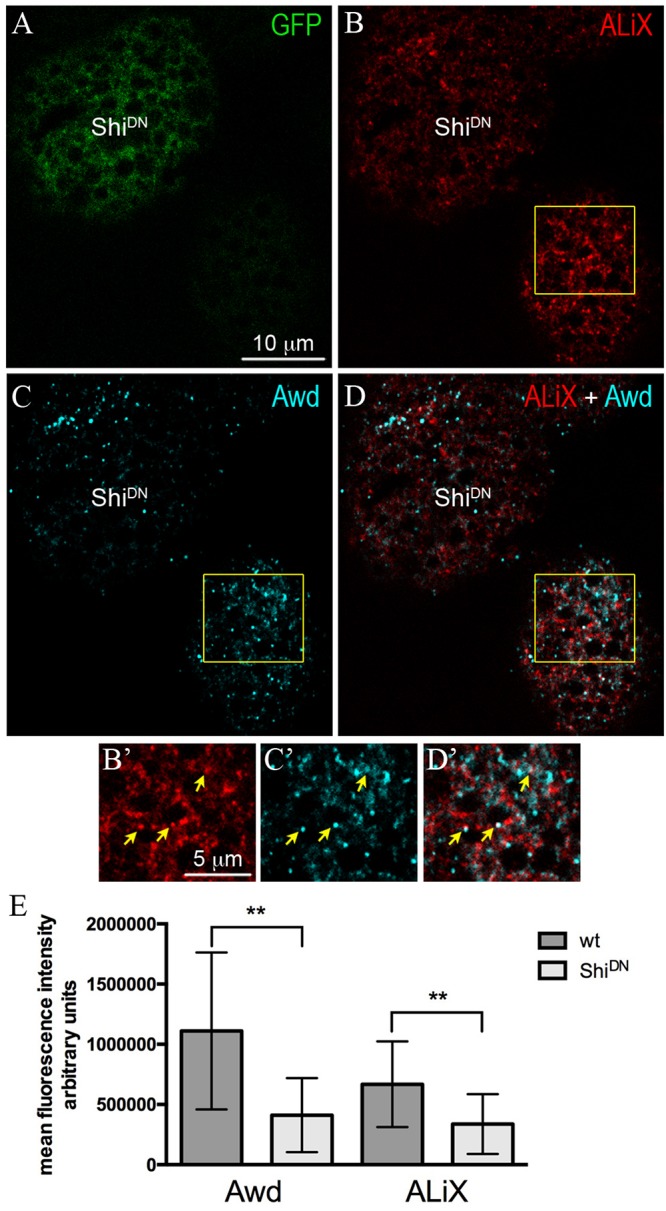
Intracellular distribution of ALiX and Awd in adipocytes expressing Shi^DN^. **(A)** Confocal microscopy analysis of fat body containing single-cell flip-out clone (GFP positive, green) over expressing Shi^DN^. **(B)** Staining for ALiX (red). **(C)** Staining for Awd (cyan). **(D)** merge of **B** and **C** signals. **(B’**–**D’)** Magnified view of the wild type adipocyte (yellow box in **B**–**D**). Yellow arrows point to colocalization of ALiX and Awd signals. **(E)** A significant decrease of Awd (*p* = 0.0076) and ALiX (*p* = 0.0013) signals is detectable in Shi^DN^ adipocytes. Graphs represent mean ± SD amount of Awd and ALiX in arbitrary units; *n* = 7; ^∗∗^*p* < 0.01 (two-tailed, paired *t-*test).

ALiX is an early-acting ESCRT factor that plays a key role in the assembly of ESCRT machinery. Besides its role in nucleating ESCRT–III complex, ALiX also acts in concentrating cargoes in vesicles (Schöneberg et al., 2017). To investigate the possibility that Awd transits in ALiX-positive vesicles, we analyzed Awd and ALiX distribution in adipocytes. Co-immunolocalization of Awd and ALiX was carried out in larval fat body expressing Shi^DN^ in flp–out clones (Figure 2A). Interestingly, confocal microscopy analysis shows that Awd (Figures 2C,C’) and ALiX (Figures 2B,B’) were partially- colocalized in wild type adipocytes (Figures 2D,D’) (Pearson’s coefficient *R* = 0,293 ± 0,059; *n* = 5). Furthermore, Shi-defective adipocytes show a significant lowering of the intracellular level of both ALiX and Awd, in comparison with the intracellular level of each protein detectable in the surrounding wild type cells (Figure 2E). Moreover, in Shi^DN^ adipocytes the colocalization level of Awd and AliX decreases as shown by the strong reduction of correlation (Pearson’s coefficient *R* = 0,027 ± 0,013; *n* = 3).

## Discussion

We have previously shown that Awd is secreted in the hemolymph by the fat body and the block of Shi activity causes the enhancement of Awd extracellular level coupled with a reduced intracellular level (Romani et al., 2016). Since Shi is required for lysosomal/autolysosomal acidification (Fang et al., 2016), the low level of Awd in Shi^DN^ adipocytes cannot be due to an increased protein degradation. Therefore, we propose that Shi plays a key role in maintenance of Awd intra-extracellular balance. Here we show that a subpopulation of ALiX-positive vesicles partially- colocalizes with Awd. This is of particular interest since ALiX controls the intracellular traffic of multiple proteins and is frequently present at the level of EVs secreted by cells (Juan and Furthauer, 2018). Block of Shi function results in lowered intracellular level of ALiX and Awd and their loss of colocalization may suggest that Awd/ALiX-positive vesicles could exit the cell contributing to their intracellular decrease.

Our functional analysis of ESCRT complex components shows that, among the subunits analyzed, the Vps28 is involved in Awd trafficking in larval adipocytes. Vps28 is a component of the ESCRT–I complex; however, loss-of-function of the other ESCRT–I component Tsg101 did not affect the Awd intracellular level and distribution. This suggests that the ESCRT machinery is acting in a Non-canonical fashion. Previous works already showed that Non-canonical ESCRT mechanisms, involving few but not all subunits, act in EVs and exosomes biogenesis (Colombo et al., 2013; Juan and Furthauer, 2018). The long-range secretion of the Hedgehog (Hh) morphogen in the larval wing disc is also regulated by a subset of ESCRT components (Matusek et al., 2014). Conditioned medium of a cell line derived from the wing imaginal disc showed the presence of Vps28, ALiX and Vps32 components. Interestingly, these vesicles also contain Awd (Matusek et al., 2014).

Taken together these results suggest a possible involvement of ALiX and Vps28 in Awd secretion.

The partial colocalization of Awd and ALiX could be explained by taking into account two considerations. First, adipocytes are able to produce and secrete Awd protein (Romani et al., 2016). Proteins addressed to secretion have to traffic to several different intracellular compartments identified by specific proteins (Barlowe and Miller, 2013). Second, it is worth noting that Shi function requires Awd activity (Krishnan et al., 2001; Dammai et al., 2003). Therefore, we would not expect that all the Awd intracellular pool will localize in membrane compartments destined for secretion.

Intriguingly, alteration of both intracellular and extracellular NME1/2 levels were shown to have implication in cancer progression (Romani et al., 2018). To date, very little is known about the mechanisms regulating NME1/2 presence in the extracellular environment. The comprehension of the mechanism controlling the balance of Awd inside and outside the cell will be relevant to understand NME1/2 functions in physiological and pathological conditions.

## Materials and Methods

### Fly Strains

All stocks were maintained and crossed at 25°C. For details of genotypes and mosaic techniques see the Supplementary Data.

### Immunostaining

Fat bodies were dissected from 3rd instar larvae in Phosphate Buffered Saline (PBS) and then immediately fixed for 20 min in 4% formaldehyde in PBS at room temperature. For additional information on staining procedure see Supplementary Data. Fluorescent images were obtained with TCS SL Leica confocal system.

## Author Contributions

EM and MI performed the experiments. MI, TH, GG, and VC conceived and designed the experiments, and wrote the manuscript. All authors read and approved the final manuscript.

## Conflict of Interest Statement

The authors declare that the research was conducted in the absence of any commercial or financial relationships that could be construed as a potential conflict of interest.

## References

[B1] Baietti M. F. Zhang Z. Mortier E. Melchior A. Degeest G. Geeraerts A. (2012). Syndecan-syntenin-ALIX regulates the biogenesis of exosomes. *Nat. Cell Biol.* 14 677–685. 10.1038/ncb2502 22660413

[B2] Barlowe C. K. Miller E. A. (2013). Secretory protein biogenesis and traffic in the early secretory pathway. *Genetics* 193 383–410. 10.1534/genetics.112.142810 23396477 PMC3567731

[B3] Biggs J. Hersperger E. Steeg P. S. Liotta L. A. Shearn A. (1990). A *Drosophila* gene that is homologous to a mammalian gene associated with tumor metastasis codes for a nucleoside diphosphate kinase. *Cell* 63 933–940. 10.1016/0092-8674(90)90496-2 2175255

[B4] Chanut-Delalande H. Jung A. C. Lin L. Baer M. M. Bilstein A. Cabernard C. (2007). A genetic mosaic analysis with a repressible cell marker screen to identify genes involved in tracheal cell migration during *Drosophila* air sac morphogenesis. *Genetics* 176 2177–2187. 10.1534/genetics.107.073890 17603108 PMC1950623

[B5] Christ L. Raiborg C. Wenzel E. M. Campsteijn C. Stenmark H. (2017). Cellular functions and molecular mechanisms of the ESCRT membrane-scission machinery. *Trends Biochem. Sci.* 42 42–56. 10.1016/j.tibs.2016.08.016 27669649

[B6] Colombo M. Moita C. van Niel G. Kowal J. Vigneron J. Benaroch P. (2013). Analysis of ESCRT functions in exosome biogenesis, composition and secretion highlights the heterogeneity of extracellular vesicles. *J. Cell Sci.* 126(Pt 24), 5553–5565. 10.1242/jcs.128868 24105262

[B7] Dammai V. Adryan B. Lavenburg K. R. Hsu T. (2003). *Drosophila awd*, the homolog of human *nm23*, regulates FGF receptor levels and functions synergistically with shi/dynamin during tracheal development. *Genes Dev.* 17 2812–2824. 10.1101/gad.1096903 14630942 PMC280629

[B8] Eikenes A. H. Malerod L. Christensen A. L. Steen C. B. Mathieu J. Nezis I. P. (2015). ALIX and ESCRT-III coordinately control cytokinetic abscission during germline stem cell division in vivo. *PLoS Genet.* 11:e1004904. 10.1371/journal.pgen.1004904 25635693 PMC4312039

[B9] Escola J. M. Kleijmeer M. J. Stoorvogel W. Griffith J. M. Yoshie O. Geuze H. J. (1998). Selective enrichment of tetraspan proteins on the internal vesicles of multivesicular endosomes and on exosomes secreted by human B-lymphocytes. *J. Biol. Chem.* 273 20121–20127. 10.1074/jbc.273.32.20121 9685355

[B10] Fang X. Zhou J. Liu W. Duan X. Gala U. Sandoval H. (2016). Dynamin regulates autophagy by modulating lysosomal function. *J. Genet. Genomics* 43 77–86. 10.1016/j.jgg.2015.10.005 26924690

[B11] Hurley J. H. Odorizzi G. (2012). Get on the exosome bus with ALIX. *Nat. Cell Biol.* 14 654–655. 10.1038/ncb2530 22743708

[B12] Ignesti M. Barraco M. Nallamothu G. Woolworth J. A. Duchi S. Gargiulo G. (2014). Notch signaling during development requires the function of awd, the *Drosophila* homolog of human metastasis suppressor gene Nm23. *BMC Biol.* 12:12. 10.1186/1741-7007-12-12 24528630 PMC3937027

[B13] Juan T. Furthauer M. (2018). Biogenesis and function of ESCRT-dependent extracellular vesicles. *Semin. Cell Dev. Biol.* 74 66–77. 10.1016/j.semcdb.2017.08.022 28807885

[B14] Koppen T. Weckmann A. Muller S. Staubach S. Bloch W. Dohmen R. J. (2011). Proteomics analyses of microvesicles released by *Drosophila* Kc167 and S2 cells. *Proteomics* 11 4397–4410. 10.1002/pmic.201000774 21901833

[B15] Krishnan K. S. Rikhy R. Rao S. Shivalkar M. Mosko M. Narayanan R. (2001). Nucleoside diphosphate kinase, a source of GTP, is required for dynamin-dependent synaptic vesicle recycling. *Neuron* 30 197–210. 10.1016/s0896-6273(01)00273-2 11343655

[B16] Lee T. Luo L. (1999). Mosaic analysis with a repressible cell marker for studies of gene function in neuronal morphogenesis. *Neuron* 22 451–461. 10.1016/s0896-6273(00)80701-1 10197526

[B17] Lloyd T. E. Atkinson R. Wu M. N. Zhou Y. Pennetta G. Bellen H. J. (2002). Hrs regulates endosome membrane invagination and tyrosine kinase receptor signaling in *Drosophila*. *Cell* 108 261–269. 10.1016/s0092-8674(02)00611-6 11832215

[B18] Malerød L. Le Borgne R. Lie-Jensen A. Eikenes A. H. Brech A. Liestol K. (2018). Centrosomal ALIX regulates mitotic spindle orientation by modulating astral microtubule dynamics. *EMBO J.* 37:e97741. 10.15252/embj.201797741 29858227 PMC6028035

[B19] Matusek T. Wendler F. Poles S. Pizette S. D’Angelo G. Furthauer M. (2014). The ESCRT machinery regulates the secretion and long-range activity of Hedgehog. *Nature* 516 99–103. 10.1038/nature13847 25471885

[B20] Moline M. M. Southern C. Bejsovec A. (1999). Directionality of wingless protein transport influences epidermal patterning in the *Drosophila* embryo. *Development* 126 4375–4384. 10477304 10.1242/dev.126.19.4375

[B21] Nallamothu G. Woolworth J. A. Dammai V. Hsu T. (2008). *awd*, the homolog of metastasis suppressor gene *Nm23*, regulates *Drosophila* epithelial cell invasion. *Mol. Cell Biol.* 28 1964–1973. 10.1128/MCB.01743-1747 18212059 PMC2268403

[B22] Okabe-Kado J. Kasukabe T. Honma Y. (1998). Differentiation inhibitory factor Nm23 as a prognostic factor for acute myeloid leukemia. *Leuk. Lymphoma* 32 19–28. 10.3109/10428199809059243 10036998

[B23] Panakova D. Sprong H. Marois E. Thiele C. Eaton S. (2005). Lipoprotein particles are required for Hedgehog and Wingless signalling. *Nature* 435 58–65. 10.1038/nature03504 15875013

[B24] Pignoni F. Zipursky S. L. (1997). Induction of *Drosophila* eye development by decapentaplegic. *Development* 124 271–278. 9053304 10.1242/dev.124.2.271

[B25] Romani P. Duchi S. Gargiulo G. Cavaliere V. (2017). Evidence for a novel function of Awd in maintenance of genomic stability. *Sci. Rep.* 7:16820. 10.1038/s41598-017-17217-17210 29203880 PMC5714947

[B26] Romani P. Ignesti M. Gargiulo G. Hsu T. Cavaliere V. (2018). Extracellular NME proteins: a player or a bystander? *Lab. Invest.* 98 248–257. 10.1038/labinvest.2017.102 29035383

[B27] Romani P. Papi A. Ignesti M. Soccolini G. Hsu T. Gargiulo G. (2016). Dynamin controls extracellular level of Awd/Nme1 metastasis suppressor protein. *Naunyn Schmiedebergs Arch. Pharmacol.* 389 1171–1182. 10.1007/s00210-016-1268-1269 27449069

[B28] Rosengard A. M. Krutzsch H. C. Shearn A. Biggs J. R. Barker E. Margulies I. M. (1989). Reduced Nm23/Awd protein in tumour metastasis and aberrant *Drosophila* development. *Nature* 342 177–180. 10.1038/342177a0 2509941

[B29] Schöneberg J. Lee I. H. Iwasa J. H. Hurley J. H. (2017). Reverse-topology membrane scission by the ESCRT proteins. *Nat. Rev. Mol. Cell Biol.* 18 5–17. 10.1038/nrm.2016.121 27703243 PMC5198518

[B30] Stafford L. J. Vaidya K. S. Welch D. R. (2008). Metastasis suppressors genes in cancer. *Int. J. Biochem. Cell Biol.* 40 874–891. 10.1016/j.biocel.2007.12.016 18280770

[B31] Steeg P. S. Bevilacqua G. Kopper L. Thorgeirsson U. P. Talmadge J. E. Liotta L. A. (1988). Evidence for a novel gene associated with low tumor metastatic potential. *J. Natl. Cancer Inst.* 80 200–204. 10.1093/jnci/80.3.200 3346912

[B32] Steeg P. S. de la Rosa A. Flatow U. MacDonald N. J. Benedict M. Leone A. (1993). Nm23 and breast cancer metastasis. *Breast Cancer Res. Treat.* 25 175–187. 10.1007/bf00662142 8347849

[B33] Tschiedel S. Gentilini C. Lange T. Wolfel C. Wolfel T. Lennerz V. (2008). Identification of NM23-H2 as a tumour-associated antigen in chronic myeloid leukaemia. *Leukemia* 22 1542–1550. 10.1038/leu.2008.107 18496563

[B34] Vaccari T. Rusten T. E. Menut L. Nezis I. P. Brech A. Stenmark H. (2009). Comparative analysis of ESCRT-I. *J. Cell Sci.* 122(Pt 14), 2413–2423. 10.1242/jcs.046391 19571114 PMC2704878

[B35] van Niel G. D’Angelo G. Raposo G. (2018). Shedding light on the cell biology of extracellular vesicles. *Nat. Rev. Mol. Cell Biol.* 19 213–228. 10.1038/nrm.2017.125 29339798

[B36] van Noesel M. M. Versteeg R. (2004). Pediatric neuroblastomas: genetic and epigenetic ’danse macabre’. *Gene* 325 1–15. 10.1016/j.gene.2003.09.042 14697505

[B37] Woolworth J. A. Nallamothu G. Hsu T. (2009). The *Drosophila* metastasis suppressor gene *Nm23* homolog, *awd*, regulates epithelial integrity during oogenesis. *Mol. Cell Biol.* 29 4679–4690. 10.1128/MCB.00297-299 19581292 PMC2725718

[B38] Wubbolts R. Leckie R. S. Veenhuizen P. T. Schwarzmann G. Mobius W. Hoernschemeyer J. (2003). Proteomic and biochemical analyses of human B cell-derived exosomes. *J. Biol. Chem.* 278 10963–10972. 10.1074/jbc.M207550200 12519789

