# Supplementary material for: Vps28 Is Involved in the Intracellular Trafficking of Awd, the Drosophila Homolog of NME1/2
Source: Front Physiol. 2019 Aug 2;10:983. doi: 10.3389/fphys.2019.00983 (PMC6687847; doi:10.3389/fphys.2019.00983)
Supplement: Supplementary file 1 [file Data_Sheet_1.docx]

Supplementary Material

- The ESCRT machinery is involved in the intracellular trafficking of Awd, the *Drosophila* homologue of NME1/2

**Elisa Mezzofanti, Marilena Ignesti, Giuseppe Gargiulo, Valeria Cavaliere***

*** Correspondence:** Corresponding Author: [valeria.cavaliere@unibo.it](mailto:valeria.cavaliere@unibo.it)

# Supplementary Data

**Fly strains and Genetics**

Flies were maintained on a standard cornmeal/yeast/agar medium at 25°C, unless otherwise stated.

The following lines were obtained from Bloomington: #4779, #7, #5137, #4775, #5822, #5191, #5192, #5135, #9917, #55813, #23650. The following mutant stocks were obtained from T. Vaccari: *FRT^42D^, vps28^A2^* - *FRT^40A^, hrs^D28^, stam^2L2896^* - *FRT^80B^, tsg101^2^* - *FRT^82B^, vps22^ZZ13^* - *FRT^82B^, vps2^PP6^*. The transgenic line carrying the *UAS-CD63:GFP* transgene was obtained from S. Eaton.

To obtain MARCM clones progeny was submitted to a heat shock at 0-4 hours AED (After Egg Deposition) for 1 hour at 37°C. At 120 hours AED larvae were selected and processed for immunostaining. To induce flp-out clones, mated females were allowed to lay eggs in a 4 hours time window. The progeny was heat shocked 48 hours AED for 8 minutes at 37°C and then it was let to develop at 25°C for a total of 120 hours AED at 25°C. To obtain flp-out clones expressing Shi^DN^, progeny was shifted from 25°C to 31°C at 48 hours AED and allowed to develop for additional 72 hours at 31°C.

The exact genotypes corresponding to the Figures 1-2 and Supplementary Figures S1-S3 are given in the Table 1.

**Immunostaining**

Fat bodies fixed in 4% paraformaldehyde were washed three times for 5 minutes in 1x PBS. After 1 hour incubation in 0,3% Triton X100 in PBS, the fat bodies were washed three times in 1x PBS for 5 minutes. Tissue was then blocked in 0,3% Triton X100 in PBS supplemented with 2% BSA for 15 minutes and incubated at 4°C with primary antibodies diluted in 0,3% Triton X100 in PBS supplemented with 2% BSA. After three washes in 0,3% Triton X100 in PBS, 15 minutes each, the tissue was blocked in 0,3% Triton X100 in PBS supplemented with 2% BSA for 15 minutes. Then, the sample were incubated with fluorescence-tagged secondary antibodies diluted PBS supplemented with 2% BSA for 2 hours at room temperature and, after three washes in 0,3% Triton X100 in PBS, they were mounted in Fluoromount G (Electron Microscopy Science). TCS SL Leica confocal system was used for image analysis. Experiments were performed at least 2 times with comparable results. For each experimental condition a minimum of 10 adipocytes were analyzed. The images were assembled using the Adobe Photoshop software. The following antibodies and dilutions were used: anti-Awd (1:2000) (Dammai et al., 2003); anti-Hrs (1:30, 27-4-5, DSHB); anti-ALiX (1:100) (Tsuda et al., 2006); CY3-anti-rabbit (1:2000, Invitrogen); Dylight 649-anti-rabbit (1:500, Jackson); CY3-anti-mouse (1:1000, Jackson); Dylight 647-anti-mouse (1:500, Jackson).

**Colocalization and statistical analysis**

Colocalization analysis was performed in wild type and Shi^DN^ adipocytes. The selected confocal images were processed through Adobe Photoshop CS4 software to adjust threshold and exclude background staining. Thresholded images were processed with the CDA (Confined Displacement Algorithm) (Ramirez et al., 2010) plugin of ImageJ to obtain the Pearson’s coefficient (from +1=complete correlation, to -1=anti-correlation with 0=no correlation) (Zinchuk and Zinchuk, 2008). The values were then analysed by using GraphPad Prism 6 software. The Pearson’s correlation coefficient was reported in the text as mean and standard deviation. n=number of analyzed adipocytes of each genotype.

**Fluorescence intensity quantification and statistical analysis**

Fluorescence intensities have been measured by using the Image J software. Cells were outlined and the Integrated Density (ID) and the area of selections were measured. For background normalization, the mean grey value was measured for five regions selected outside the cell and then the Corrected Total Cell Fluorescence (CTCF) was calculated by using the following equation: CTCF = ID – (Area of selected cell x mean fluorescence of background readings).

CTCFs have been estimated for statistical significance using a two-tailed distribution paired Student’s *t*-test with Prism software. The results are reported in the graph as means with standard deviation. n=number of analyzed adipocytes of each genotype.

**Table 1 List of genotypes**

| **Figure 1** | **(A)** *w; His2Av-mRFP1 (III.1)*  **(C-I)** *yw, hs-flp, tub-Gal4, UAS-nGFP/+; FRT^42D^, vps28^A2^/FRT^42D^, tub-Gal80* |
| --- | --- |
| **Figure 2** | *yw, hs-flp/act>CD2>Gal4; UAS-nGFP/+; TM3, UAS-shi^K44A^/+* |
| **Supplementary Figure 1** | **(A-C’)** *yw, hs-flp, tub-Gal4, UAS-nGFP/+; FRT^40A^, hrs^D28^, stam^2L2896^/FRT^40A^, tub-Gal80* |
|  | **(D-G’)** *yw, hs-flp, tub-Gal4, UAS-nGFP/+; FRT^80B^, tsg101^2^/FRT^80B^, tub-Gal80* |
| **Supplementary Figure 2** | **(A-G)** *w, tub-Gal4, UAS-nGFP, hs-flp/+; FRT^82B^, vps22^ZZ13^/FRT^82B^, tub-Gal80* |
|  | **(H-J)** *yw, hs-flp, UAS-mGFP, tub-Gal4/+; FRT^82B^, vps2^PP6^/FRT^82B^, tub-Gal80* |
| **Supplementary Figure 3** | **(A-C’)** *yw, act>CD2>Gal4/+; UAS-CD63:GFP/+; UAS-mRFP/hs-flp* |
|  | **(D-F’)***yw, act>CD2>Gal4/+; UAS-CD63:GFP/+; TM3, UAS-shi^K44A^/hs-flp* |

# Supplementary Figures

**
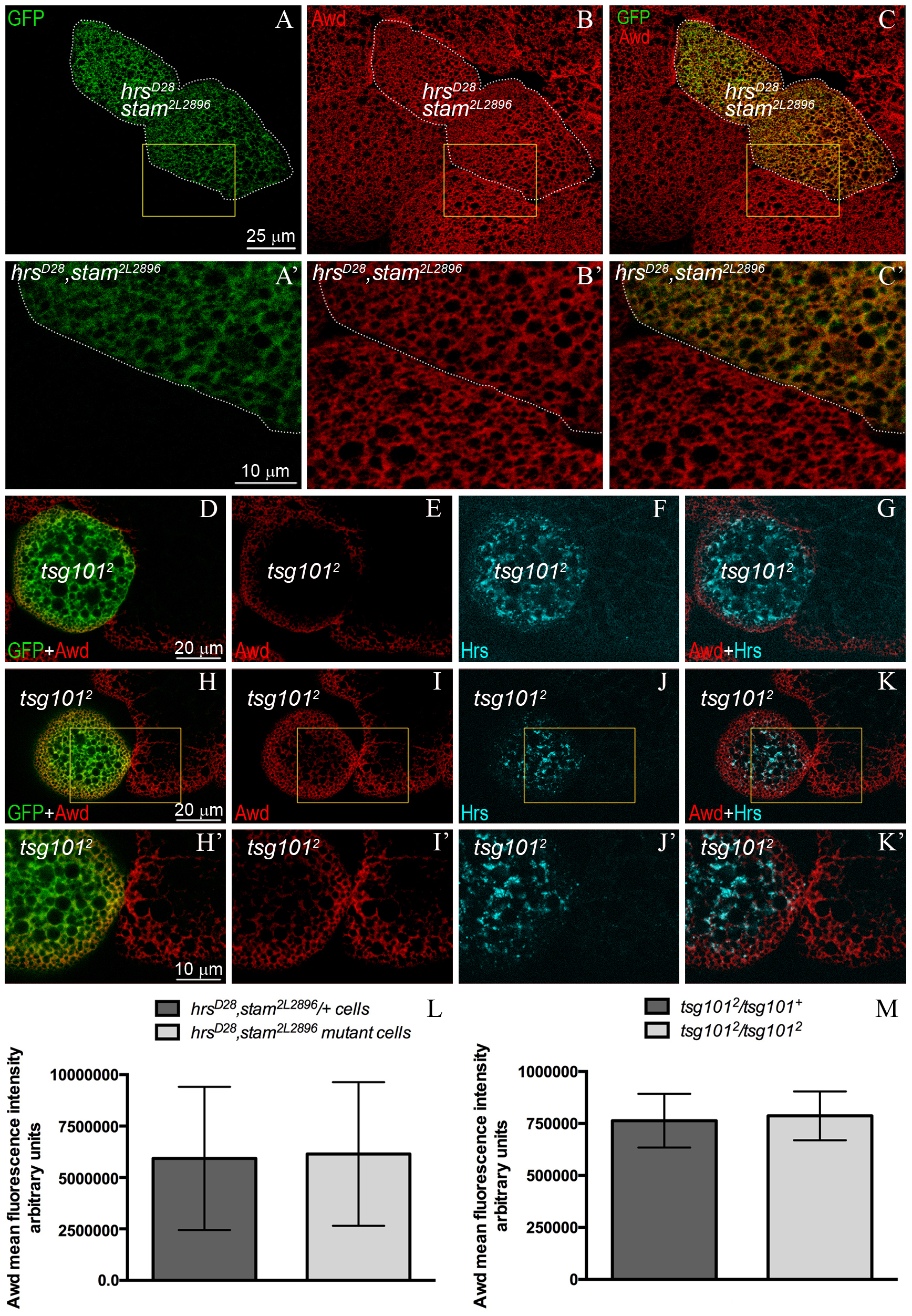
**

**Supplementary Figure S1 |** Awd protein distribution in ESCRT-0 and Tsg101 mutants.

Confocal microscopy analysis of *hrs^D28^, stam^2L2896^* **(A**-**C’)** and *tsg101^2^* **(D**-**K’)** MARCM clones of adipocytes (marked by GFP expression, green) stained for Awd (red) and Hrs (cyan). The images in **(H**-**K)** are cortical sections of adipocytes. (**D**-**G**) Confocal section taken in a less cortical plane to show that the cells in **(H**-**K)** are not detached from each other. The yellow boxes in **(A**-**C)** and **(H**-**K)** outline the regions magnified in (**A’**-**C’**) and (**H’**-**K’**), respectively. (**L**) Quantification of fluorescence intensity of Awd signal in *hrs^D28^, stam^2L2896^* and wild type adipocytes. p = 0.7624 (two-tailed, paired *t-*test). (**M**) Quantification of fluorescence intensity of Awd signal in *tsg101^2^* and wild type adipocytes. p = 0.5722 (two-tailed, paired *t-*test). Graphs represent mean ± SD amount of Awd in arbitrary units; n = 3.

**
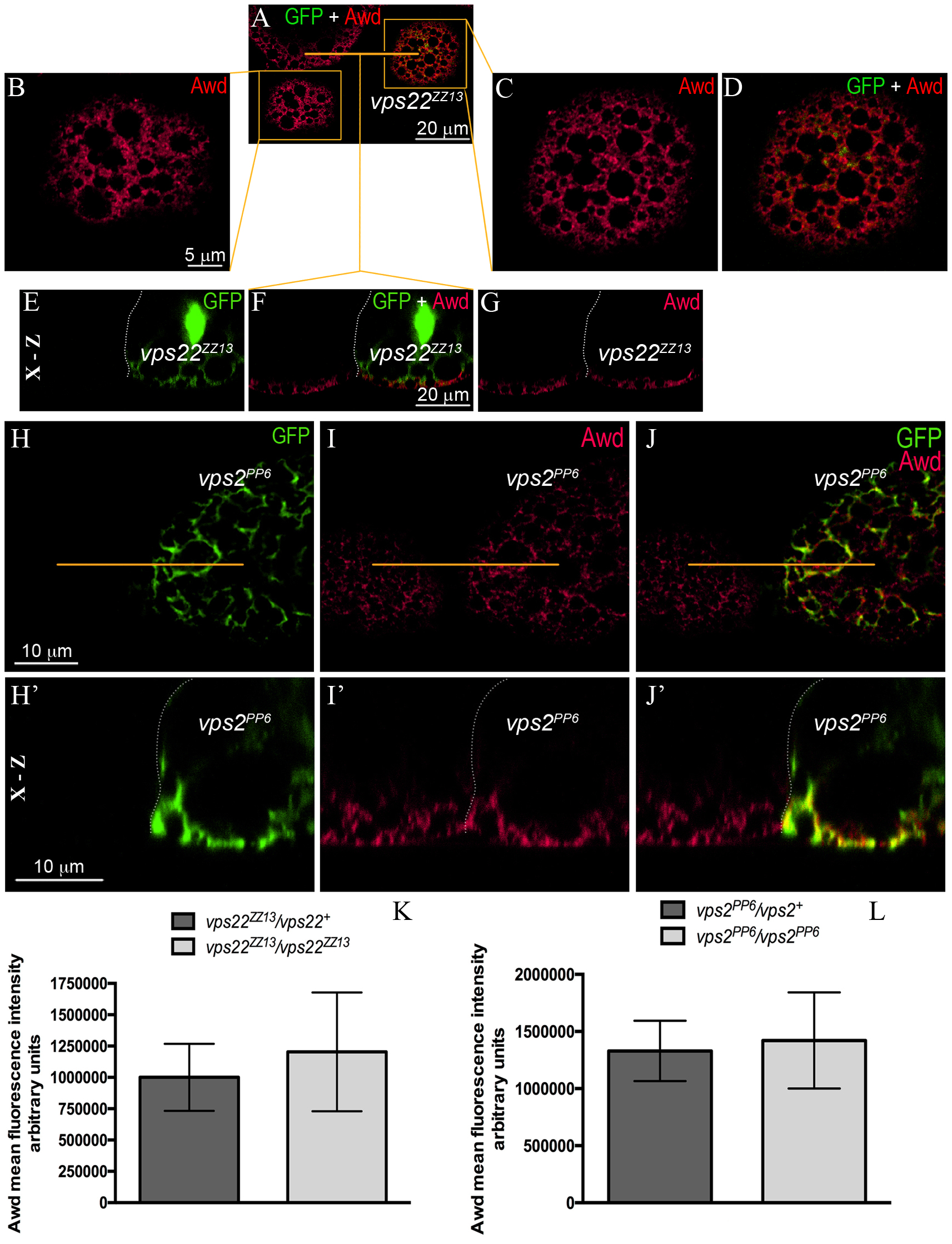
**

**Supplementary Figure S2 |**Awd protein distribution in mutants belonging to ESCRT-II and –III complexes.

Confocal microscopy analysis of *vps22^ZZ13^* (**A**-**G**) and *vps2^PP6^* (**H**-**J’**) MARCM clones of adipocytes marked by GFP expression (green) stained for Awd (red). The orange boxes in (**A**) outline the regions magnified in (**B**-**D**). (**E**-**G**) and (**H’**-**J’**) x-z sections through the planes indicated by the orange line in (**A**) and (**H**-**J**), respectively. (**K**) Quantification of fluorescence intensity of Awd signal in *vps22^ZZ13^* and wild type adipocytes. p = 0.2349 (two-tailed, paired *t-*test) (**L**) Quantification of fluorescence intensity of Awd signal in *vps2^PP6^* and wild type adipocytes. p = 0.4178 (two-tailed, paired *t-*test). Graphs represent mean ± SD amount of Awd in arbitrary units; n = 3.

**
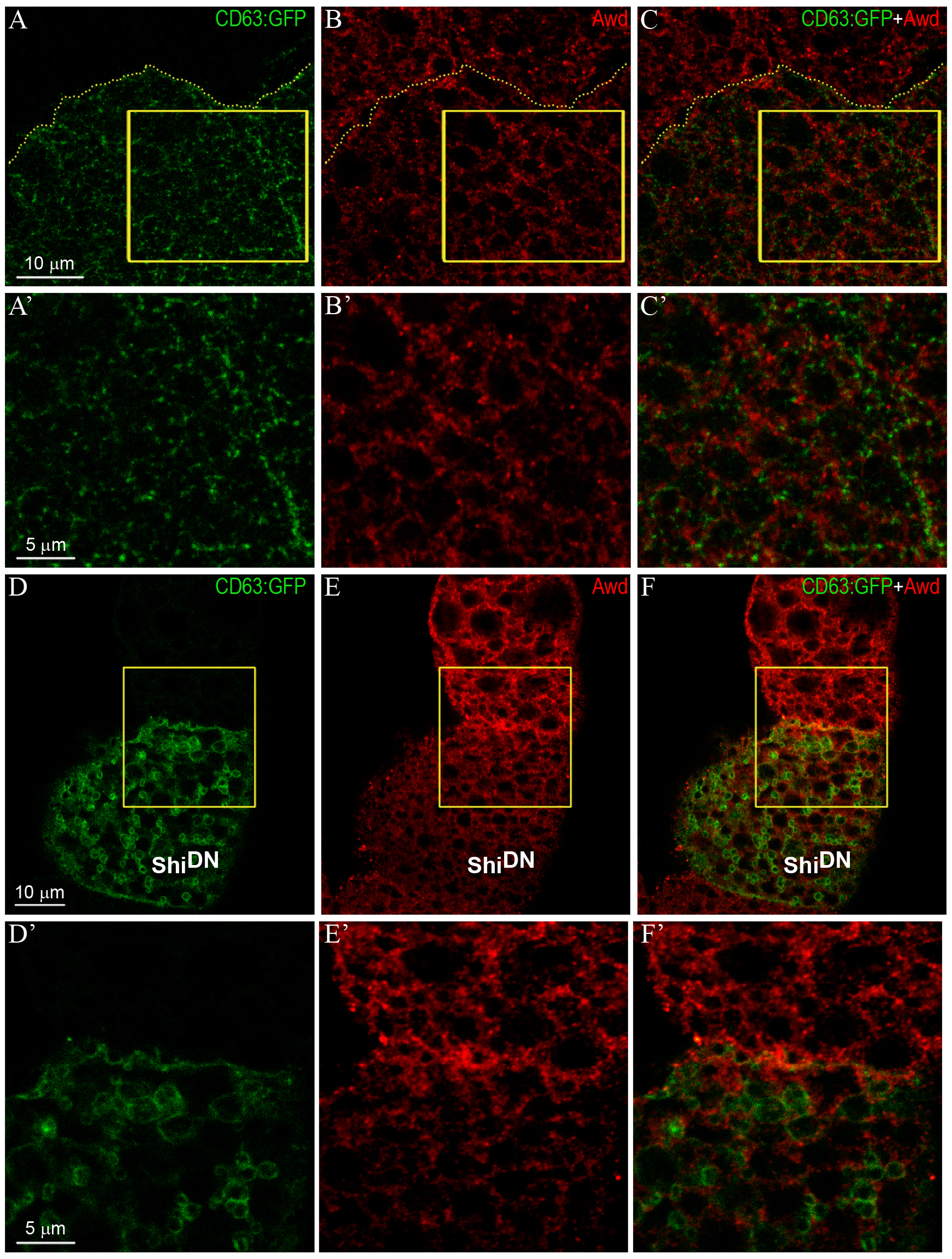
**

**Supplementary Figure S3 |** Awd and CD63:GFP distribution in wild type and Shi^DN^ mutant adipocytes.

Confocal microscopy analysis of flp-out clones expressing CD63:GFP (green) (**A**-**C**) and flp-out clones co-expressing CD63:GFP (green) together with Shi^DN^ (**D**-**F’**) stained for Awd (red). The yellow boxes in **(A-C)** and **(D-F)** outline the regions magnified in (**A’**-**C’**) and (**D’**-**F’**), respectively.

**Supplementary References**

Dammai, V., Adryan, B., Lavenburg, K.R., and Hsu, T. (2003). *Drosophila awd*, the homolog of human *nm23*, regulates FGF receptor levels and functions synergistically with *shi/dynamin* during tracheal development. *Genes Dev* 17(22)**,** 2812-2824. doi: 10.1101/gad.1096903.

Ramirez, O., Garcia, A., Rojas, R., Couve, A., and Hartel, S. (2010). Confined displacement algorithm determines true and random colocalization in fluorescence microscopy. *J Microsc* 239(3)**,** 173-183. doi: 10.1111/j.1365-2818.2010.03369.x.

Tsuda, M., Seong, K.H., and Aigaki, T. (2006). POSH, a scaffold protein for JNK signaling, binds to ALG-2 and ALIX in Drosophila. *FEBS Lett* 580(13)**,** 3296-3300. doi: 10.1016/j.febslet.2006.05.005.

Zinchuk, V., and Zinchuk, O. (2008). Quantitative colocalization analysis of confocal fluorescence microscopy images. *Curr Protoc Cell Biol* Chapter 4**,** Unit 4 19. doi: 10.1002/0471143030.cb0419s39.
